# Systematic rare variant analyses identify *RAB32* as a susceptibility gene for familial Parkinson’s disease

**DOI:** 10.1038/s41588-024-01787-7

**Published:** 2024-06-10

**Authors:** Paul J. Hop, Dongbing Lai, Pamela J. Keagle, Desiree M. Baron, Brendan J. Kenna, Maarten Kooyman, Shankaracharya, Cheryl Halter, Letizia Straniero, Rosanna Asselta, Salvatore Bonvegna, Alexandra I. Soto-Beasley, Project MinE ALS Sequencing Consortium, Zbigniew K. Wszolek, Ryan J. Uitti, Ioannis Ugo Isaias, Gianni Pezzoli, Nicola Ticozzi, Owen A. Ross, Jan H. Veldink, Tatiana M. Foroud, Kevin P. Kenna, John E. Landers

**Affiliations:** 1 Department of Translational Neuroscience, UMC Utrecht Brain Center, University Medical Center Utrecht, Utrecht, the Netherlands; 2 Department of Neurology, UMC Utrecht Brain Center, University Medical Center Utrecht, Utrecht, the Netherlands; 3 Department of Medical and Molecular Genetics, Indiana University School of Medicine, Indianapolis, IN USA; 4 Department of Neurology, UMass Chan Medical School, Worcester, MA USA; 5 Department of Biomedical Sciences, Humanitas University, Milan, Italy; 6 IRCCS Humanitas Research Hospital, Rozzano, Milan, Italy; 7 Parkinson Institute, ASST Gaetano Pini-CTO, Milan, Italy; 8 Department of Neuroscience, Mayo Clinic, Jacksonville, FL USA; 9 Department of Neurology, Mayo Clinic, Jacksonville, FL USA; 10 Department of Neurology, University Hospital of Würzburg and Julius Maximilian University of Würzburg, Würzburg, Germany; 11 Fondazione Grigioni per il Morbo di Parkinson, Milan, Italy; 12 Department of Neurology–Stroke Unit and Laboratory of Neuroscience, Istituto Auxologico Italiano IRCCS, Milan, Italy; 13 Department of Pathophysiology and Transplantation, ‘Dino Ferrari’ Center, Università degli Studi di Milano, Milan, Italy; 14 Department of Clinical Genomics, Mayo Clinic, Jacksonville, FL USA

**Keywords:** Genome-wide association studies, Parkinson's disease, Genomics

## Abstract

Despite substantial progress, causal variants are identified only for a minority of familial Parkinson’s disease (PD) cases, leaving high-risk pathogenic variants unidentified^1,2^. To identify such variants, we uniformly processed exome sequencing data of 2,184 index familial PD cases and 69,775 controls. Exome-wide analyses converged on *RAB32* as a novel PD gene identifying c.213C > G/p.S71R as a high-risk variant presenting in ~0.7% of familial PD cases while observed in only 0.004% of controls (odds ratio of 65.5). This variant was confirmed in all cases via Sanger sequencing and segregated with PD in three families. *RAB32* encodes a small GTPase known to interact with LRRK2 (refs. ^3,4^). Functional analyses showed that RAB32 S71R increases LRRK2 kinase activity, as indicated by increased autophosphorylation of LRRK2 S1292. Here our results implicate mutant RAB32 in a key pathological mechanism in PD—LRRK2 kinase activity^5–7^—and thus provide novel insights into the mechanistic connections between RAB family biology, LRRK2 and PD risk.

## Abstract (web summary)

Analysis of exome sequencing data identifies a missense variant in RAB32 associated with high risk of familial Parkinson’s disease. Functional studies show that this risk variant increases LRRK2 kinase activity.

## Main

Approximately 10–15% of patients with Parkinson’s disease (PD) are classified as ‘familial cases’, a designation conventionally restricted to patients known to have a first degree relative also affected by the disorder^8,9^. The identification of causal rare variants in familial PD has contributed enormously to our current understanding of the disease, ultimately yielding drug targets, biomarkers and key insights into disease mechanisms^1,10–12^. Currently, seven genes have been classified as definitely associated with familial PD^2,13^. The discovery of these familial PD genes has been achieved through various family-based study designs, including linkage analysis, homozygosity mapping and segregation filtering^1,10,12^. However, in many cases, conventional family-based study designs are insufficient to identify causal variants due to a combination of genetic heterogeneity across families, reduced penetrance and limited sample size within families. Rare variant association testing methods, such as gene burden analysis, provide an alternative strategy for discovering rare genetic risk factors^14^. Instead of leveraging familial structure, these methods leverage case–control differences in cumulative rare variant frequencies. This approach has recently been used to study rare genetic variation in idiopathic PD^15^, but thus far, targeted analyses of familial PD cases have not been performed. In previous work, we demonstrated substantial power gains for disease gene discovery in amyotrophic lateral sclerosis by restricting rare variant association testing to familial cases and controls^16–18^. The rationale of selecting familial cases is to increase sensitivity by enriching for patients carrying rare variants of large effect. In this Letter, we apply this strategy to familial PD, conducting to our knowledge the largest genetic analysis of familial PD so far.

**Extended Data Fig. 1:**
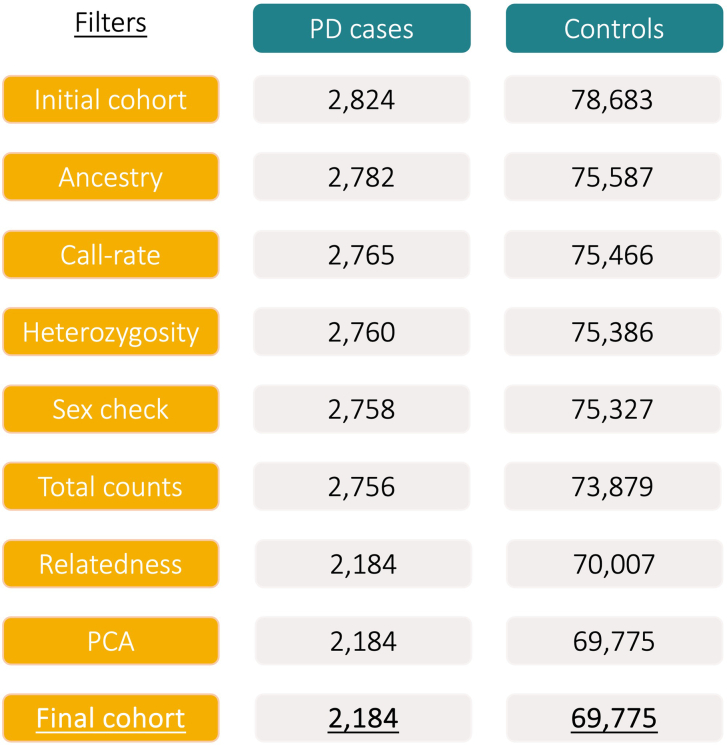
Overview of the sample quality control (QC) steps leading to the final cohort of 2,184 individuals with PD and 69,775 controls. For each filter, the number of samples retained after applying the filter is shown. First, samples of broad European ancestry were retained, then samples were excluded in case of low call rate (<0.9), outlying heterozygosity rate (*F* < − 0.1 or *F* > 0.1), genetically predicted sex that did not match reported sex, a deviant number of SNVs, INDELs, singletons or outlying Ti/Tv or SNV/INDEL ratio, ≤2^nd^ degree relatedness (one of each pair is kept) or outlying values on the first five principal components.

**Extended Data Fig. 2:**
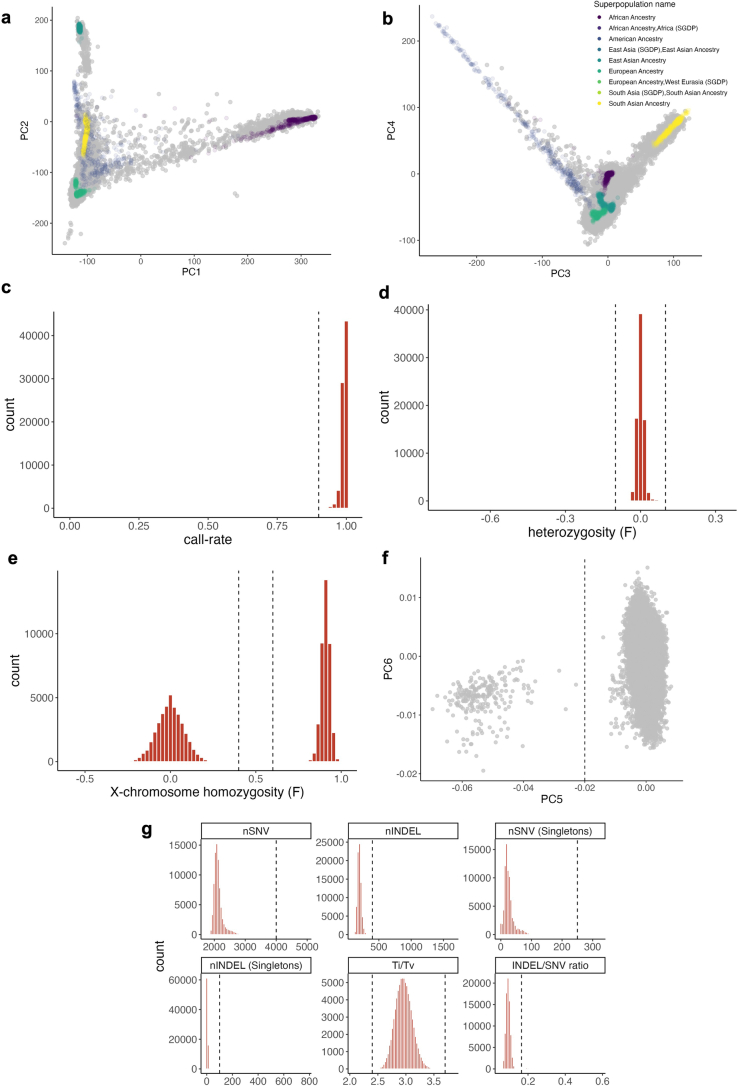
Sample QC. **a**,**b**, Projection of all samples onto PCA coordinates of a reference ancestry space comprising 1000 Genomes samples. Grey dots indicate the 81,507 samples included in this study. Colored dots indicate the 1000 Genomes samples, colored by superpopulation label, onto which the study samples were projected. **c**, Sample call-rate distribution. Samples with a call-rate < 0.9 were excluded. **d**, Heterozygosity. Samples with *F* < − 0.1 or F > 0.1 were excluded. **e**, X-chromosome homozygosity. Samples with ambiguous sex (0.4 < *F* < 0.6) or where genetically predicted sex did not match reported sex were excluded (*F* < 0.4 = female; *F* > 0.6 = male). **f**, Principal component analysis (PCA). A distinct cluster was identified on the fifth principal component, samples with PC5 < −0.02 were excluded. **g**, Total variant count distributions. Samples were excluded if nSNV > 4000, nINDEL > 400, nSNV (Singletons) > 250, nINDEL (Singletons) > 100, Ti/Tv ratio < 2.4 or > 3.7 or INDEL/SNV ratio > 0.165.

**Extended Data Fig. 3:**
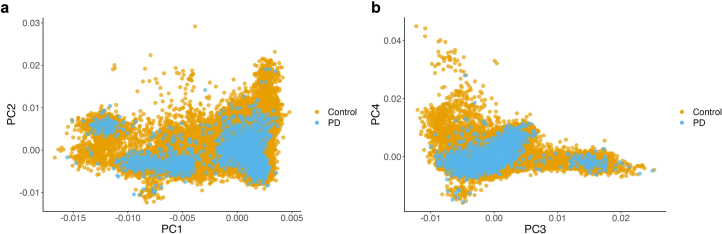
Principal components analysis of final analysis cohort consisting of 2,184 individuals with PD and 69,775 controls. **a**, Principal component 1 and 2. **b**, Principal component 3 and 4.

**Extended Data Fig. 4:**
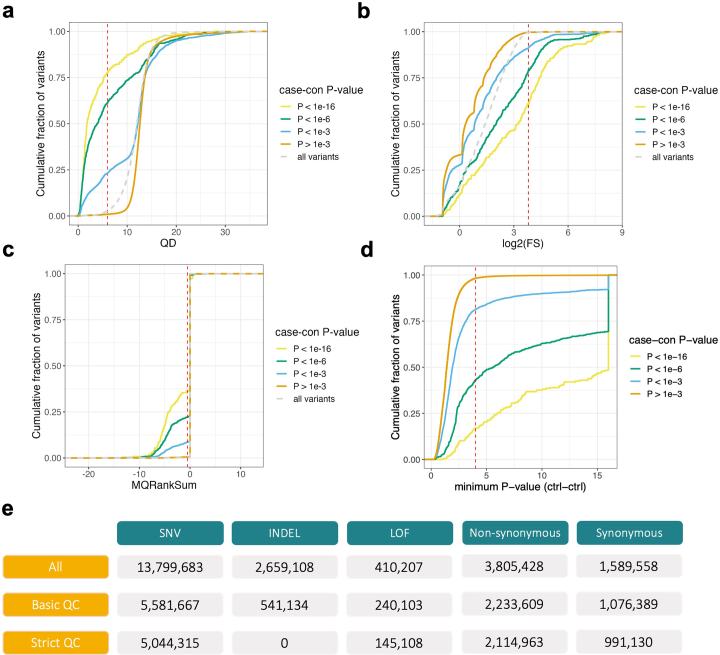
Variant quality control. **a**–**d**, Calibration of quality score thresholds for variants that pass basic quality control (VQSR-pass, per-supercohort call-rate > 0.9, HWE equilibrium *P*-value in controls > 0.0001). Plots show the cumulative fraction of variants split by case-control *P*-value (two-tailed; Firth’s logistic regression). Thresholds were set at inflection points so that the majority of variants were retained while excluding a high proportion of variants strongly associated with case-control status. The following thresholds were applied: QD ≥ 6, MQRankSum ≥ −0.5, FS ≤ 14, and control-control minimum *P*-value ≥ 0.0001 (two-tailed; Firth’s logistic regression). **e**, Overview of variant QC steps. The first row shows the total number of called variants, the second row shows the number of variants passing basic QC, and the third row shows the number of variants passing strict QC. The strict QC variant filters were used in all analyses presented in this manuscript.

We combined sequencing data from 16 cohorts, including both whole-exome and whole-genome sequencing (WGS) data, comprising a total of 2,824 individuals with familial PD and 78,683 controls. We uniformly processed all sequence data, including alignment to the Genome Reference Consortium human build 38 (GRCh38) reference genome and joint variant calling^19^. We retained individuals of predominantly European ancestry who met quality control criteria (2,756 cases and 73,879 controls) and then retained unrelated individuals, resulting in an association cohort consisting of 2,184 index familial PD cases (one affected individual per family) and 69,775 controls (Extended Data Figs. 1–3). Within this cohort, we identified 5,044,315 variants that passed strict quality control, out of which 2,114,963 were predicted to be nonsynonymous (Extended Data Fig. 4).

**Fig. 1:**
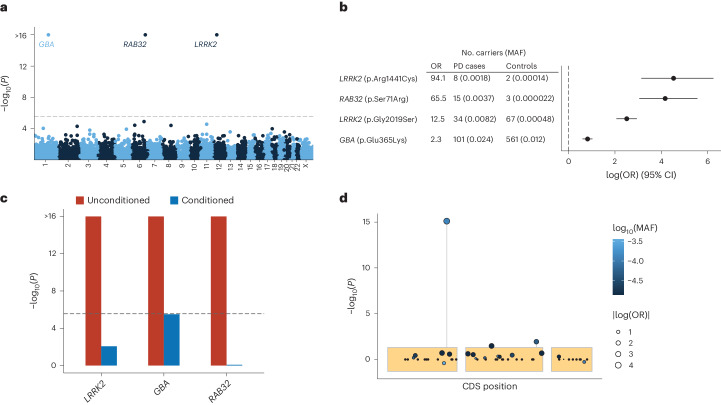
Rare nonsynonymous gene-based and single-variant analyses in 2,184 PD cases and 69,775 controls identify *RAB32*, *LRRK2* and *GBA.* **a**, The *y* axis shows the gene-based associations from the ACAT omnibus test including Firth’s logistic regression, SKAT and ACAT-V (two-tailed −log_10_(*P* value)) plotted against genomic coordinates on the *x* axis (GRCh38). The dashed line indicates the exome-wide significance threshold (*P* = 2.7 × 10^−6^). **b**, Estimated odds ratios (OR) (log transformed, *x* axis) and 95% confidence intervals (CI) of the exome-wide significant single variants obtained from Firth’s logistic regression. **c**, Conditional analyses (MAF <0.05) of significant genes using the ACAT omnibus test including Firth’s logistic regression, SKAT and ACAT-V (two-tailed). In red are the unconditioned gene associations and in blue are the conditioned gene associations, adjusted for the significant single variants within the respective gene (p.E365K in *GBA*, p.G2019S and p.R1441C in *LRRK2*, and p.S71R in *RAB32*). **d**, Single-variant associations in *RAB32* estimated using Firth’s logistic regression (*y* axis; two-tailed −log_10_(*P* value)), plotted against coding sequence positions (CDS; *x* axis).

**Extended Data Fig. 5:**
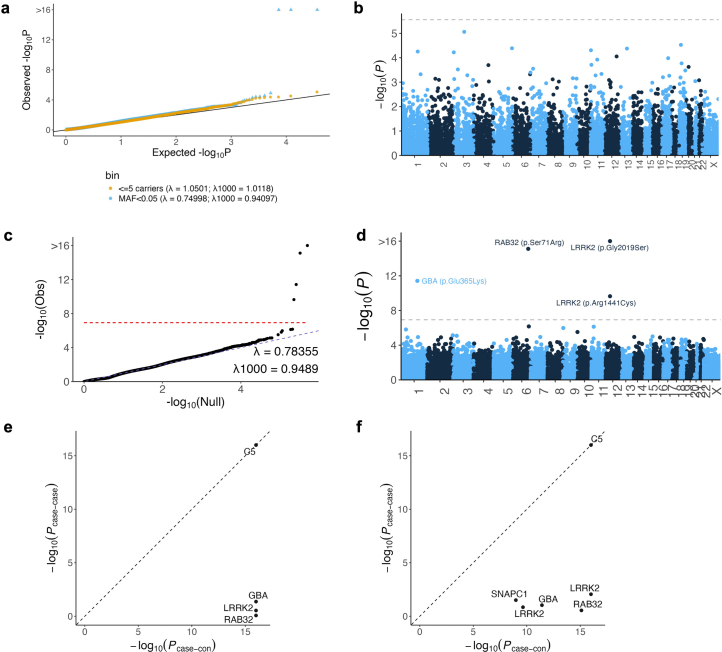
Rare variant analyses. **a**, Quantile-quantile (qq) plot of observed gene two-tailed −log_10_ (*P*-values) versus expected two-tailed −log_10_ (*P*-values) under the null model. Gene-based associations of low-frequency (MAF < 0.05) variants were estimated using the ACAT omnibus test including Firth’s logistic regression, SKAT and ACAT-V. Gene-based associations of ultra-rare variants (≤5 carriers) were estimated using Firth’s logistic regression. **b**, Gene-based analysis of ultra-rare (≤5 carriers) non-synonymous variants. The *y*-axis shows the gene-based associations (two-tailed −log_10_(*P*-value)) plotted against genomic coordinates on the *x*-axis (GRCh38). The dashed line indicates exome-wide significance (*P* = 2.74 × 10^−6^). **c**, Quantile-quantile (qq) plot of observed single variant two-tailed −log_10_ (*P*-values) versus expected two-tailed −log_10_ (*P*-values) under the null model. The red dotted line indicates the exome-wide significance threshold (*P* = 1.17 × 10^−7^). λ indicates the observed genomic inflation factor, λ_1000_ indicates the genomic inflation factor for an equivalent study of 1,000 cases and 1,000 controls. **d**, The *y*-axis shows the single variant associations estimated using Firth’s logistic regression (two-tailed −log_10_(*P*-value)) plotted against genomic coordinates on the *x*-axis (GRCh38). The dashed line indicates the exome-wide significance threshold (*P* = 1.17 × 10^−7^). **e**,**f**, Significant genes and single variants were screened for technical biases arising from different sequencing centers by testing for an association with sequencing center (*n* = 1,048 and *n* = 1,136) among PD cases. The *y*-axis shows the two-tailed −log_10_ (*P*-values) from this case-case analysis compared to the two-tailed −log_10_ (*P*-values) from the case-control analysis. Genes and single variants were excluded if *P*_case-case_ ≤ *P*_case-con_. In both gene-based (**e**) and single-variant analyses (**f**), technical bias was observed in the *C5* gene. This was caused by one variant (*C5*; c.3127 C > A), of which all 44 carriers were sequenced in the same sequencing center.

To identify genes associated with familial PD, we performed gene-based analyses for both low-frequency (minor allele frequency (MAF) <0.05) and ultrarare (≤5 carriers) nonsynonymous variants using an omnibus test that combines burden, sequencing kernel association test (SKAT) and the aggregated Cauchy association variant test (ACAT-V) (Methods)^20–22^. No significant disease associations were observed within the ultrarare variant category (Extended Data Fig. 5a,b); however, analyses of low-frequency variants (MAF <0.05) revealed disease associations for *LRRK2*, *GBA* and *RAB32* at exome-wide significance (*P* < 2.7 × 10^−6^; Fig. 1a and Supplementary Data 1). Concurrently, we conducted exome-wide single-variant analyses, identifying four exome-wide significant variants in *LRRK2*, *GBA* and *RAB32* (Fig. 1b, Extended Data Fig. 5c,d and Supplementary Data 2). Among the four significant variants, three had been previously implicated in PD, including c.1093G > A/p.E365K in *GBA*; c.6055G > A/p.G2019S and c.4321C > T/p.R1441C in *LRRK2* (test statistics for all known PD variants are provided in Supplementary Data 2). The fourth variant, a c.213C > G/p.S71R variant of *RAB32* (odds ratio of 65.5, *P* = 7.8 × 10^−16^), represented a novel discovery. We observed no evidence of genomic inflation in any of the analyses performed (gene *λ*_1,000_ = 0.94, single variant *λ*_1,000_ = 0.95; Extended Data Fig. 5). Of note, two additional genes (*SNAPC1* and *C5*) were initially flagged as potential exome-wide significant associations but were then confirmed as technical artifacts in subsequent validation analyses (Methods, Supplementary Tables 1 and 2, Extended Data Fig. 5e,f, and Supplementary Figs. 1 and 2).

**Fig. 2:**
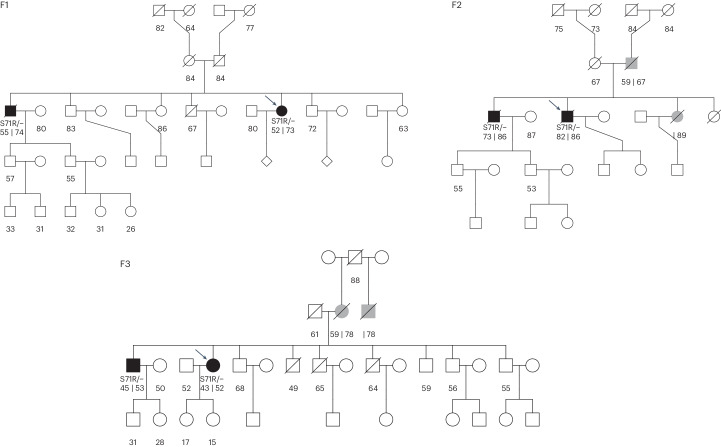
Pedigrees of families with multiple sequenced individuals. Unaffected family members are indicated by white symbols, affected family members with verified PD are indicated by black symbols and family members with nonverified or reported PD are indicated by gray symbols. For individuals with PD, the age of onset is displayed at the left and age at death or last follow-up is displayed on the right (separated by ‘|’). For nonaffected individuals, the age at death or last follow-up is displayed. Probands are indicated by arrows. Pedigrees for which only the proband was sequenced are presented in Extended Data Fig. 6.

**Extended Data Fig. 6:**
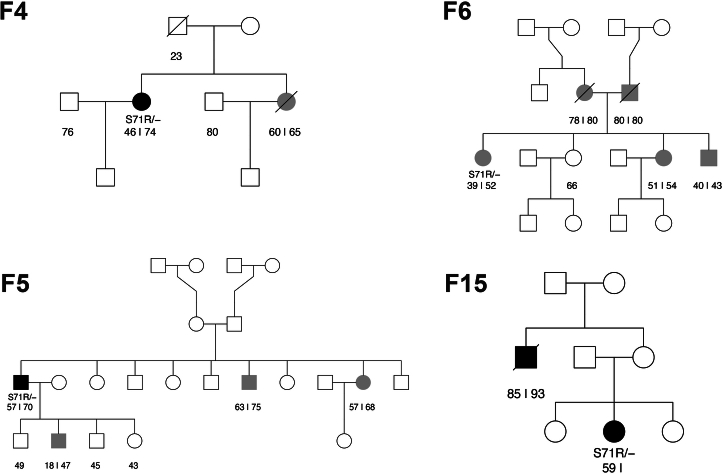
Pedigrees of families with one sequenced individual. Unaffected family members are indicated by white symbols, affected family members with verified PD are indicated by black symbols, family members with non-verified or reported PD are indicated by grey symbols. For individuals with PD, age of onset is displayed at the left and age at death or last follow-up is displayed on the right (separated by a ‘|’). For non-affected individuals, the age at death or last follow-up is displayed.

**Extended Data Fig. 7:**
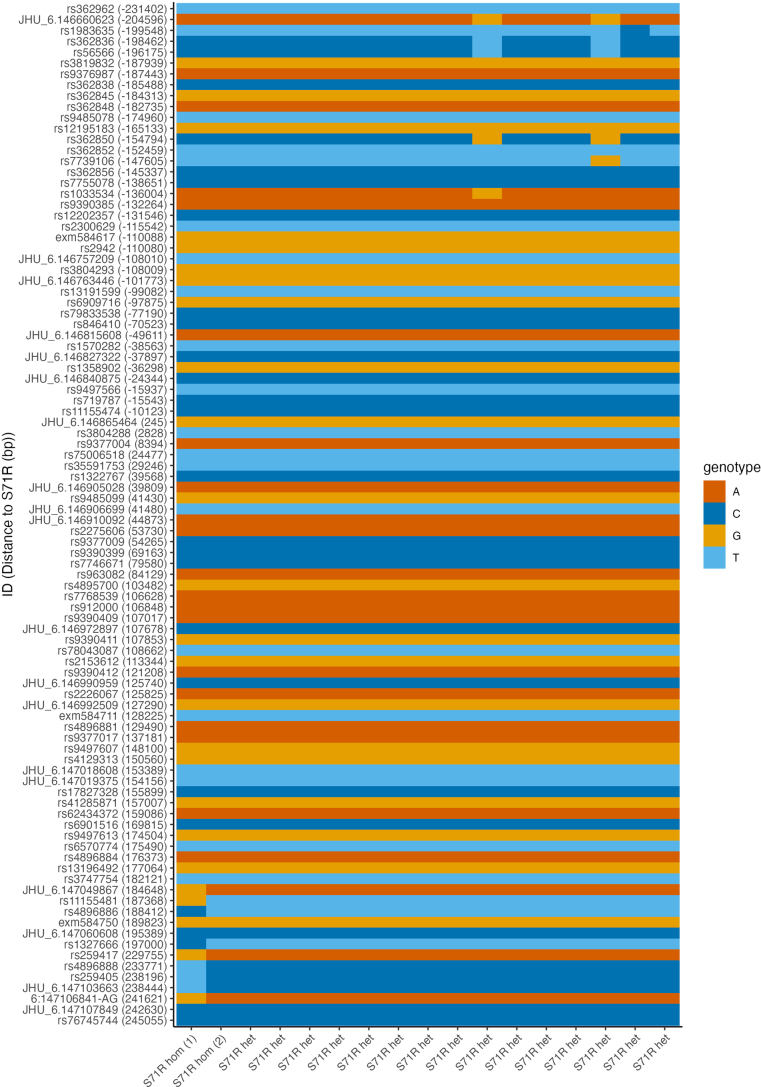
Shared haplotype for *RAB32* p.S71R. The figure shows the shared haplotype in a ± 250-kb window surrounding the p.S71R variant based on common markers on the Infinium Global Diversity Array-8 (available for 16 out of 18 p.S71R carriers). The *y*-axis shows SNP IDs with the distance to the p.S71R variant in parentheses, and the *x*-axis shows the p.S71R genotype (both alleles are shown for the homozygous carrier). The minimal common region extends from -131,546 bp downstream to 182,121 bp upstream of the p.S71R variant.

Both the gene-based and single-variant analyses converged on *RAB32* as a novel PD gene. Conditional analysis revealed that the observed *RAB32* gene association was due to the p.S71R variant (*P*_conditional_ = 0.83; Fig. 1b–d), whereas conditional analyses for *LRRK2* and *GBA* confirmed that known residual associations from additional disease relevant rare variants were detectable within the dataset (Fig. 1c; *GBA*, *P*_conditional_ = 3.1 × 10^−6^ and *LRRK2*, *P*_conditional_ = 8.7 × 10^−3^). Among 2,184 index PD cases, we identified one homozygous carrier and 15 heterozygous carriers of the *RAB32* p.S71R variant (MAF_case_ of 0.0037), while only three (heterozygous) variant carriers were identified among the 69,775 controls (MAF_ctrl_ of 0.000022). The MAF observed in the control group is similar to that reported in 44,329 non-neurological European ancestry individuals in the gnomAD (v2.1.1) reference database (MAF_gnomAD_ of 0.000023), confirming that the variant is very rare in the general population^23^. For three PD cases carrying the p.S71R variant, exome sequencing data were available for one of their affected siblings, and the variant segregated with the disease in all three families (Fig. 2 and Extended Data Fig. 6). Thus, in total, we identified 18 PD cases carrying the p.S71R variant, of which 17 were heterozygous carriers and one was a homozygous carrier (no evidence for inbreeding, *F* = −0.004). Sanger sequencing confirmed the presence of the variant in all 18 PD cases (Supplementary Table 1 and Supplementary Fig. 1). Haplotype analysis indicated that carriers shared a ~300-kb haplotype surrounding the p.S71R variant (Extended Data Fig. 7). Evaluation of other known PD variants revealed the co-occurrence of p.S71R in two carriers with *GBA* risk variants (c.1093G > A/p.E365K and c.1223C > T/p.T408M) (Extended Data Table 1).

We identified an additional four carriers among 3,380 PD cases (0.12%) in the publicly available Accelerating Medicines Partnership program for PD WGS data (https://www.amp-pd.org/), of which two were familial PD cases (out of 900; 0.22%), one was a sporadic PD case and family history was not available for the fourth case.

**Extended Data Fig. 8:**
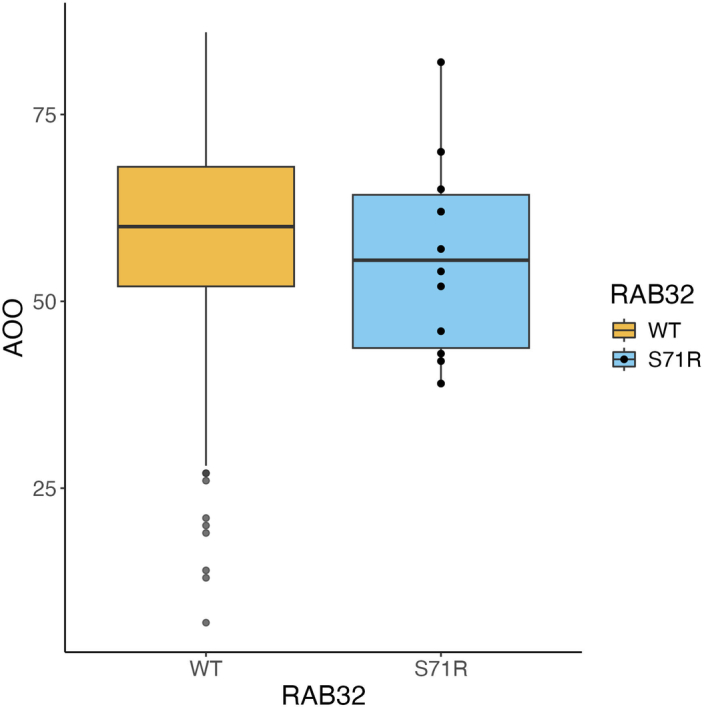
Age of onset (AOO) distribution of *RAB32* p.S71R carriers versus *RAB32* wild-type patients. Age at onset was known for 1,414 unrelated individuals. The box edges represent the interquartile range (75^th^ and 25^th^ percentile) with the horizontal line indicating the median. The whiskers extend to 1.5 times the IQR from the box edges. Individual data points beyond the ends of the whiskers are shown for RAB32 wild-type (WT); all individual data points are shown for RAB32 S71R. No statistically significant difference was observed in a linear regression adjusting for cohort (b = −2.7 years, two-tailed *P* = 0.38).

Carriers of the *RAB32* p.S71R variant had a mean age of onset of 56 years (s.d. 13; range, 39–82 years), which did not significantly differ from noncarriers (Extended Data Fig. 8 and Extended Data Table 1). The variant was observed in 11 males and 7 females, reflecting the overall sex ratio of patients in this study (~60% male). All p.S71R carriers evaluated for L-dopa response were confirmed as L-dopa responsive (15 evaluated and 3 not evaluated) and for the majority (10 out of 18), dyskinesia and/or motor fluctuations were reported. For all but one of the carriers, asymmetry was reported (16 out of 17), and the majority presented with rest tremors (11 out of 18). Moreover, postural instability, freezing of gait and/or falls were common symptoms among carriers reported in 11 out of 18 patients. Detailed case reports were available for nine patients (Supplementary Note). Among them, seven out of nine patients reported nonmotor symptoms, including, among others, hyposmia, autonomic dysfunction, sleep problems and depression. Notably, two patients had a medical history of Hashimoto’s thyroiditis. The case report of the homozygous carrier shows that the clinical presentation did not notably differ from that of heterozygote carriers. He had late-onset (61 years), L-dopa-responsive PD characterized by a right resting tremor and micrographia with motor fluctuations and dyskinesia emerging 9 years after onset. The patient passed away 12 years after the onset, with falls and mild cognitive impairment appearing 10 and 11 years after onset, respectively.

RAB32 belongs to the RAB family of small GTPases, which is composed of 70 members that play key roles in regulating intracellular vesicular transport^24^. Various members of the RAB family are known to be either substrates or regulators of the key PD protein LRRK2 (refs. ^24–26^). Most pathogenic *LRRK2* variants increase kinase activity, in turn resulting in increased phosphorylation of several RAB family members^25,27,28^. Interestingly, while there is no evidence supporting phosphorylation of RAB32 by LRRK2, RAB32 has been shown to interact with LRRK2 through the N-terminal Armadillo domain of LRRK2, and may regulate its subcellular localization^3,4^. Moreover, overexpression of RAB29, a member of the RAB32 subfamily, has been shown to activate LRRK2, recruiting it to the *trans*-Golgi network and increasing its kinase activity^26^.

**Fig. 3:**
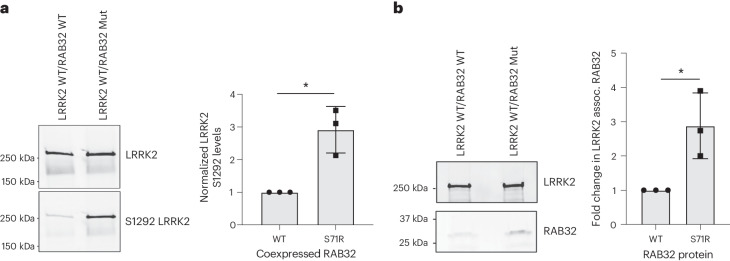
RAB32 S71R triggers increased LRRK2 kinase activity. Western blot analyses of cotransfections in HEK293 cells, where Myc-LRRK2 WT was introduced alongside either HA-RAB32 WT or HA-RAB32 S71R. The bar plots show mean ± s.d. **a**, LRRK2 S1292 phosphorylation; *n* = 3 biological replicates are shown (two-tailed one-sample *t*-test, *P* = 0.02; Extended Data Fig. 9). **b**, LRRK2 and RAB32 coimmunoprecipitation; *n* = 3 biological replicates are shown (two-tailed one-sample *t*-test, *P* = 0.034; Extended Data Fig. 9). *P < 0.05. Full-length blots are provided as source data. Mut, mutant. Source data

**Fig. 4:**
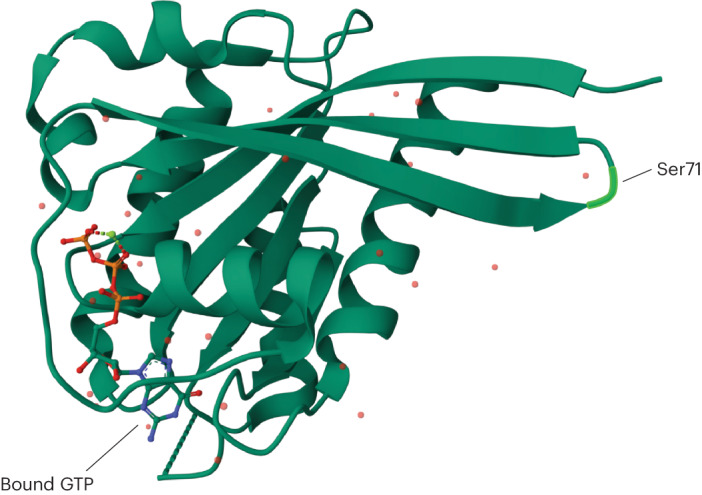
Three-dimensional protein structure of RAB32 highlighting the phosphorylation site at Ser71. Crystal structure of uncomplexed Rab32 in the active GTP-bound state at 2.13 Å resolution^4,34^.

**Extended Data Fig. 9:**
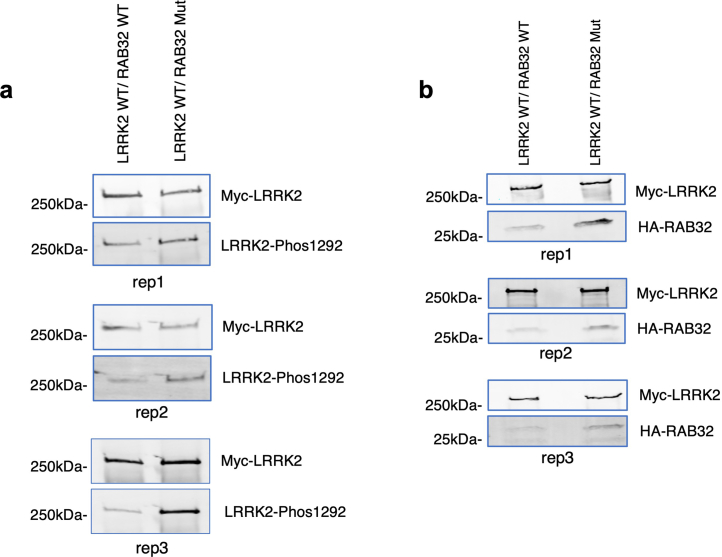
Western blots for all biological replicates. **a**,**b**, Western blots for all three biological replicates of LRRK2 S1292 phosphorylation (**a**) and LRRK2 and RAB32 co-immunoprecipitation (**b**). Full-length blots are provided as Source Data. Source data

Given the established interaction between LRRK2 and RAB32, along with the closely related RAB29 functioning as a LRRK2 activator, we hypothesized that RAB32 S71R results in increased LRRK2 activation. To this end, we performed cotransfections in HEK293 cells, introducing Myc-LRRK2 wild type (WT) alongside either HA-RAB32 WT or HA-RAB32 S71R. Autophosphorylation of S1292 provides a known in vivo readout of LRRK2 kinase activity, as well as a biomarker for the development of parkinsonian phenotypes in patients carrying *LRRK2* mutations^27,29,30^. As anticipated, we observed a significant increase in LRRK2 S1292 phosphorylation levels in cells expressing mutant RAB32 versus WT RAB32 (approximately threefold increase, *P* = 0.02; Fig. 3a and Extended Data Fig. 9). In keeping with this, we also observed a threefold increase in the interaction between LRRK2 and RAB32 through coimmunoprecipitation and western blotting (*P* = 0.034; Fig. 3b and Extended Data Fig. 9). Finally, previous phosphoproteomic analysis indicated that Ser71 constitutes the only known phosphorylation site on the RAB32 protein (Fig. 4)^31^. The S71R variant probably interferes with this phosphorylation event, and this may provide an explanation for why the S71R variant alone was found to exhibit a robust association with PD risk. Taken together, these results support a model where the S71R variant selectively impacts the functioning of RAB32 in a manner that increases its interaction with LRRK2 and, through this, increases LRRK2 activity.

In conclusion, we present, to our knowledge, the largest genetic association study of familial PD so far, identifying p.S71R in *RAB32* as a novel risk variant and substantiating that the toxicity of this variant is most probably mediated through enhancement of LRRK2 activity. Importantly, substantial evidence points toward increased LRRK2 kinase activity as the primary pathological mechanism in LRRK2 PD and, to some extent, idiopathic PD, and consequently, reduction of LRRK2 activity has emerged as a major therapeutic focus^5–7^. Future research is needed to determine the mechanism through which RAB32 S71R increases LRRK2 activity. Of particular interest is the possibility that RAB32 S71R leads to tetramerization of LRRK2, similar to RAB29^32^, which was shown to increase LRRK2 autophosphorylation in contrast to WT RAB32. Our study underscores that, as with prior work in amyotrophic lateral sclerosis^16–18^, the combination of familial case ascertainment and a sizable control cohort enables the identification of low-frequency variants that were previously limited to detection by linkage analyses. This is evidenced first and foremost by the discovery of *RAB32* p.S71R, but also by the recovery of known ultrarare PD variants that had not previously been detectable in genome-wide case–control analyses (*LRRK2* p.R1441C, MAF_ctrl_ 0.000014, *P* = 2.27 × 10^−10^). Interestingly, our findings are corroborated by a recent study^33^, which discovered that *RAB32* p.S71R segregates with PD in three families, not overlapping with our study. Consistent with our results, this parallel study demonstrates that RAB32 S71R enhances LRRK2 autophosphorylation, as well as RAB10 phosphorylation. This independent study provides complementary evidence that validates our findings, thus firmly establishing *RAB32* p.S71R as a causal factor in familial PD. Finally, the identification of *RAB32* as a familial PD gene provides new insight into the important mechanistic connections between RAB family biology, LRRK2 and PD risk.

## Methods

### Cohorts

We received approval for this study from the institutional review boards of the participating centers, and written informed consent was obtained from all patients (consent for research). Informed consent was obtained for the publication of individual case reports. The discovery cohort included 2,824 patients with PD and 78,683 controls, totaling 81,507 study participants, of which 10,270 were subjected to WGS and 71,237 to whole-exome sequencing (WXS). One thousand six hundred familial PD cases were from the PROGENI study, which recruited multiplex PD families primarily through a living affected sibling pair. Subsequently, ascertainment was loosened to include PD probands having a positive family history of PD in a first-degree relative, who was not required to be part of the study. All individuals completed an in-person evaluation that included parts II and III of the Unified Parkinson Disease Rating Scale (Lang and Fahn 1989) and a diagnostic checklist that implemented the UK PD Brain Bank inclusion and exclusion criteria. Responses on the diagnostic checklist were then used to classify study subjects as having verified PD or nonverified PD. A total of 783 familial PD cases were recruited at the Mayo clinic, patients were included who fulfilled the clinical diagnostic criteria of PD^35^. A total of 441 familial PD cases were from the Parkinson Institute Biobank; diagnosis of PD was made according to the UK Brain Bank criteria^35^. Control cohorts included 7,323 samples from the National Heart, Lung, and Blood Institute Trans-Omics for Precision Medicine research program^36^; 49,981 samples from the UK Biobank^37^; 2,805 samples from Project MinE^38^; 342 samples from the New York Genome Center’s amyotrophic lateral sclerosis (ALS) consortium and 18,232 samples from database of Genotypes and Phenotypes (included studies and accession number are listed in the ‘Data availability’ section)^39^.

### Sequencing

All raw sequencing data was aligned to the GRCh38 reference genome using Burrow–Wheeler aligner maximal exact match (BWA–MEM; v.2.2.1)^40^ according to the pipeline described by Regier et al.^41^ (implementation can be found at GitHub^42^ and Zenodo^43^). Joint genotyping was performed using a uniform pipeline according to the Genome Analysis Toolkit (GATK) best practices (v. 4.2.6.1)^19^. Genotype calls with a quality score <20 were set to missing, variant calls supported by uninformative reads were excluded and multiallelic variants were split into biallelic variants. Male genotypes in nonpseudoautosomal regions on chromosome X were coded as 0 or 1 (according to 0 or 1 allele copies).

### Variant annotation

Variants were annotated using snpEff^44^, dbscSNV^45^ and Ensembl Release 105 gene models^46^. Variants were classified as loss-of-function when predicted by snpEff to have a high impact (including nonsense mutations, splice acceptor/donors and frameshift mutations) or predicted as potentially splice altering by dbscSNV (‘ada’ or ‘rf’ score >0.7). Variants were classified as having moderate impact when predicted as such by snpEff (including missense mutations and inframe deletions). For each gene, the impact of a variant was determined by its most severe consequence across protein-coding transcripts.

### Sample quality control

Ancestry was estimated by projecting all samples on a reference ancestry space comprising 1000 Genomes samples using the LASER software (v2.04)^47^. We retained individuals of predominantly European ancestry (Extended Data Figs. 1 and 2). We then excluded samples with low genotype call rates (<0.9), discordant sex or deviating heterozygosity (*F* < −0.1 or *F* > 0.1). These metrics were calculated in a set of autosomal variants meeting the following criteria: call rate >0.9 in each supercohort (WGS, WXS_UKB_ and WXS_other_), MAF >0.01, and for sex inference, heterozygosity, relatedness and principal component analysis (PCA) variants were additionally filtered on the basis of Hardy–Weinberg equilibrium (*P* < 0.001) and pruned if in linkage disequilibrium (*r*^2^ < 0.5, window size of 50, step 5; additionally, high linkage disequilibrium regions were excluded before PCA^48^). We then excluded samples based on a high exome-wide number of single-nucleotide variants (SNVs), insertions/deletions (INDELs), singletons, high INDEL/SNV ratio or deviating Ti/Tv ratio (thresholds listed in Extended Data Fig. 2). Sample duplicates and relatives up to and including the second degree were identified using the KING software^49^. An unrelated sample set was generated by first excluding samples with five or more relations, followed by iteratively excluding individuals with the highest number of relations, resolving ties by prioritizing (in order) PD over controls and WGS over WXS samples. PCA was performed on the unrelated sample set using fastPCA as implemented in PLINK2 (ref. ^50^). A distinct cluster was identified on the fourth and fifth principal component, consisting of an Amish population, which was excluded since the cluster contained only controls (Extended Data Fig. 2).

### Variant quality control

First, GATK variant quality score recalibration (VQSR) was applied to all variants using the training data and annotations as recommended by the GATK best practices^19^. Variants were excluded if they did not pass variant quality score recalibration, their genotyping rate was <0.9 in any of the supercohorts (WGS, WXS_UKB_ and WXS_other_) or if they did not pass the Hardy–Weinberg equilibrium test in control subjects (*P* < 0.0001). We then additionally excluded variants with subpar quality scores, variants located in regions showing signs of batch effects and we retained only SNVs. Potential batch effects were identified by testing whether variant minor allele counts were associated with cohort membership within control subjects. Firth’s logistic regression was used to perform these control–control analyses, adjusting for sex and four principal components. This procedure was repeated for each cohort (that is, number 1 represents individual in respective cohort, number 0 represents otherwise). In total, 16 control versus control cohort comparisons were tested (including all WGS controls versus all WXS controls). The minimum *P* value across these analyses was used as a metric to identify variants associating with probable batch effects. The stringency of various standard variant quality control filters was then increased to eliminate variants exhibiting batch associated calling bias while maintaining maximal sensitivity for unbiased variant calls (Extended Data Fig. 4). In total 5,044,315 variants passed quality control, including 2,114,963 nonsynonymous variants (Extended Data Fig. 4).

### Single-variant analyses

Single-variant analyses were performed for all nonsynonymous variants with MAF <0.05 and a minor allele count of at least five (425,827 variants). For each variant, we tested for an association between PD status and minor allele count using Firth’s logistic regression adjusting for sex, the first ten principal components and the total number of rare synonymous variants in each individual^20^. All tests were two sided and Bonferroni correction was employed to correct for multiple testing. Significant single-variant associations were screened for potential technical biases arising from different sequencing centers among cases by using the same procedure employed in the control–control analyses. Variants where *P*_case–case_ < *P*_case–control_ were flagged as being potentially driven by technical variation (Extended Data Fig. 5). In addition, we performed Sanger sequencing to validate novel identified variants (see ‘Sanger sequencing’ section). Haplotypes were inferred using Beagle (v.5.4) in single-nucleotide polymorphism (SNP) array data (Infinium Global Diversity Array-8)^51^.

### Gene-based analyses

Gene-based rare variant analyses were performed for 18,242 protein-coding genes, including either low frequency (MAF <0.05) or ultrarare (≤5 carriers) nonsynonymous variants (loss-of-function or moderate impact). For ultrarare variants, we performed burden tests by testing for an association between PD status and the aggregate effect of minor alleles observed per sample per gene. For low-frequency variants (MAF <0.05), we additionally performed SKAT and ACAT-V tests^22,52^. To account for the unbalanced case–control ratio, burden tests were performed using Firth’s logistic regression^20^, the robust version of SKAT was employed^21^ and we adapted ACAT-V so that Firth’s logistic regression is used to perform the burden and single-variant tests that underlie ACAT-V^53^. Test statistics across the three statistical tests were combined using ACAT, which is designed to combine potentially correlated test statistics^22^. In each of the tests, we included sex, the first ten principal components and the total number of qualifying synonymous variants in each individual as covariates. Tests were retained when there were at least five variant carriers across the gene.

We performed conditional analyses to assess to what extent gene-based associations were driven by significant variants identified in the single-variant analysis. Conditional gene-based analyses were performed as described above, excluding and adjusting for the significant single variants within the respective gene.

### Sanger sequencing

Sanger sequencing of *RAB32* exon 1 and *SNAPC1* c.1073-2A > T was performed using touchdown PCR (30 cycles with annealing temperature starting at 65 °C and decreasing 0.5 °C per cycle, followed by 15 cycles with an annealing temperature of 65 °C) using primers generated with M13 tails. Amplification was performed using AmpliTaqGold 360 Master mix (Thermo Fisher Scientific, 4398876). Patient DNA samples were provided after extraction from whole blood. PCR products were visualized on a 2% agarose gel using gel electrophoresis and subsequently purified by incubation with Exonuclease I (New England Biolabs, M0293L) and shrimp alkaline phosphatase (Sigma-Aldrich, GEE70092X). Following purification, all PCR products were sequenced at the MGH Massachusetts General Hospital DNA Core Facility. Sequence analysis was performed using the PHRED/PHRAP/Consed software suite (http://www.phrap.org/) and variations in the sequences were identified with the Polyphred v6.15 software^54^. The PCR and sequencing primers are listed in Supplementary Tables 1 and 2. Primer set 1a in Supplementary Table 1 is for one sample (sample 17 in Extended Data Table 1) that has a primer sitting on top of the common SNP, rs41285869. New primers were designed to avoid this common SNP as a false homozygous variant was visualized as compared with the exome sequencing results.

### Immunoprecipitation

#### Immortalized cell culture

HEK293 cells (ATCC) were maintained at 37 °C with 5% CO_2_ in Dulbecco’s modified Eagle medium (Invitrogen) supplemented with 10% (vol/vol) heat inactivated fetal bovine serum (MediaTech), 2 mM l-glutamine (Gibco) and 1% (vol/vol) penicillin and streptomycin solution (Gibco).

#### Plasmids used for this study

The 2xMyc-LRRK2 WT plasmid was acquired from Addgene (number 25361) and confirmed via whole plasmid sequencing at Massachusetts General Hospital. The HA-RAB32 constructs, WT and S71R, were made and sequenced by Genescript. All constructs were prepped with a Maxi prep kit (Qiagen) following the manufacturer’s instructions.

#### Immunoprecipitation and western blot

HEK293 cells were transfected with Lipofectamine 2000 (Invitrogen) according to the manufacturer’s instructions using a DNA:Lipo ratio of 3 μg:6 μl per well of a six-well plate. For cotransfections, the DNA amount was divided at a 2:1 ratio for LRRK2 WT:RAB32 constructs. A total of 48 h posttransfection, the lysates were collected in denaturing hypotonic lysis buffer^55^ and protein concentration determined by Pierce BCA Protein Assay (Thermo Fisher). The 1× PBS + 0.1% Tween 20 washed Myc–antibody conjugated magnetic beads were combined with lysate in a 1 μl:5 μg ratio before rotating the immunoprecipitation overnight at 4 °C. The beads were washed with denaturing wash buffer^55^ before protein elution by boiling in 2× loading sample buffer (Boston Bioproducts). Approximately 50% of the eluate from each condition was run on a 4–20% gradient gel (Bio-Rad) and transferred to a polyvinylidene difluoride membrane (Bio-Rad) on the high molecular weight setting of a Trans-blot Turbo system (Bio-Rad). The membranes were processed as described previously^56^. Primary antibodies were used as follows: 1:2,000 for rabbit anti-HA (DSHB, catalog number anti-HA rRb-IgG), 1:1,000 mouse anti-Myc (BioLegend, catalog number 626802) and 1:300 Phos-LRRK2 S1292 (Abcam, catalog number 203181). Blots were visualized with an Odyssey Infrared Imager (LiCor, model 9120).

### Reporting summary

Further information on research design is available in the Nature Portfolio Reporting Summary linked to this article.

## Online content

Any methods, additional references, Nature Portfolio reporting summaries, source data, extended data, supplementary information, acknowledgements, peer review information; details of author contributions and competing interests; and statements of data and code availability are available at 10.1038/s41588-024-01787-7.

### Supplementary information

Supplementary Information Supplementary Tables 1 and 2, Figs. 1 and 2, and Note.

Reporting Summary

Supplementary Data 1 Summary statistics for all gene-based association analyses.

Supplementary Data 2 Summary statistics for all single variant analyses.

### Source data

Source Data Fig. 3 Uncropped western blots.

Source Data Extended Data Fig. 9 Uncropped western blots.

## Figures and Tables

**Extended Data Table 1:**
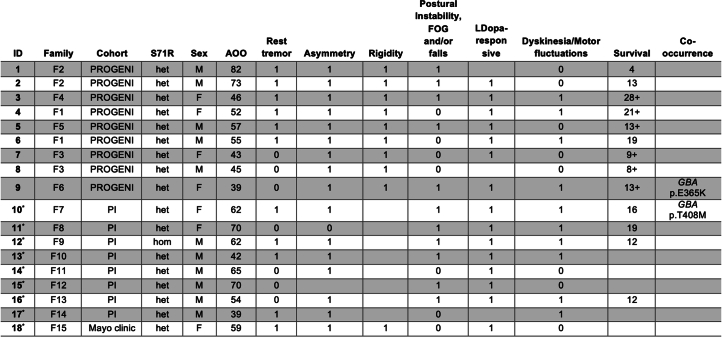
Clinical characteristics for carriers of the *RAB32* p.S71R variant

Individuals for which detailed case reports are available are indicated by an asterisk (Supplementary Note). ‘1’ indicates that the symptom is present, ‘0’ indicates the symptom was not present, blank indicates unknown. A ‘+’ in the Survival field indicates that the individual was alive at last follow-up. Abbreviations: PI, Parkinson Institute; AOO, age of onset; FOG, freezing of gait.

## Data Availability

Exome sequencing data from the case-cohort is available under dbGaP study phs001004 or are available via the corresponding author subject to the data sharing terms of the respective cohorts. Project MinE data are available upon request (https://www.projectmine.com/research/data-sharing/). dbGAP datasets used are available under the following accession numbers: Alzheimer’s Disease Sequencing Project (ADSP) (phs000572), Autism Sequencing Consortium (ASC) (phs000298), Sweden-Schizophrenia Population-Based Case–Control Exome Sequencing (phs000473), Inflammatory Bowel Disease Exome Sequencing Study (phs001076), Myocardial Infarction Genetics Exome Sequencing Consortium: Ottawa Heart Study (phs000806), Myocardial Infarction Genetics Exome Sequencing Consortium: Malmo Diet and Cancer Study (phs001101), Myocardial Infarction Genetics Exome Sequencing Consortium: University of Leicester (phs001000), Myocardial Infarction Genetics Exome Sequencing Consortium: Italian Atherosclerosis Thrombosis and Vascular Biology (phs000814), NHLBI GO-ESP: Women’s Health Initiative Exome Sequencing Project (WHI)—WHISP (phs000281), Building on GWAS for NHLBI diseases: the US CHARGE Consortium (CHARGE-S)—CHS (phs000667), Building on GWAS for NHLBI Diseases: the US CHARGE Consortium (CHARGE-S)—ARIC (phs000668), Building on GWAS for NHLBI diseases: the US CHARGE Consortium (CHARGE-S)—FHS (phs000651), NHLBI GO-ESP Family Studies: Idiopathic Bronchiectasis (phs000518), NHLBI GO-ESP: Family Studies (Hematological Cancers) (phs000632), NHLBI GO-ESP: Family Studies (familial atrial fibrillation) (phs000362), NHLBI GO-ESP: Heart Cohorts Exome Sequencing Project (ARIC) (phs000398), NHLBI GO-ESP: Heart Cohorts Exome Sequencing Project (CHS) (phs000400), NHLBI GO-ESP: Heart Cohorts Exome Sequencing Project (FHS) (phs000401), NHLBI GO-ESP: Lung Cohorts Exome Sequencing Project (asthma) (phs000422), NHLBI GO-ESP: Lung Cohorts Exome Sequencing Project (COPDGene) (phs000296), GO-ESP: Family Studies (thoracic aortic aneurysms leading to acute aortic dissections) (phs000347), NHLBI TOPMed: Genomic Activities such as Whole Genome Sequencing and Related Phenotypes in the Framingham Heart Study (phs000974), NHLBI TOPMed: Genetics of Cardiometabolic Health in the Amish (phs000956), NHLBI TOPMed: Genetic Epidemiology of COPD (COPDGene) (phs000951), NHLBI TOPMed: The Vanderbilt Atrial Fibrillation Registry (VU_AF) (phs001032), NHLBI TOPMed: Cleveland Clinic Atrial Fibrillation (CCAF) Study (phs001189), NHLBI TOPMed: Partners HealthCare Biobank (phs001024), NHLBI TOPMed—NHGRI CCDG: Massachusetts General Hospital (MGH) Atrial Fibrillation Study (phs001062), NHLBI TOPMed: Novel Risk Factors for the Development of Atrial Fibrillation in Women (phs001040), NHLBI TOPMed—NHGRI CCDG: The Vanderbilt AF Ablation Registry (phs000997), NHLBI TOPMed: Heart and Vascular Health Study (HVH) (phs000993), NHLBI TOPMed—NHGRI CCDG: Atherosclerosis Risk in Communities (ARIC) (phs001211), NHLBI TOPMed: The Genetics and Epidemiology of Asthma in Barbados (phs001143), NHLBI TOPMed: Women’s Health Initiative (WHI) (phs001237), NHLBI TOPMed: Whole Genome Sequencing of Venous Thromboembolism (WGS of VTE) (phs001402), and NHLBI TOPMed: Trans-Omics for Precision Medicine (TOPMed) Whole Genome Sequencing Project—Cardiovascular Health Study (phs001368). Additional data used include: GATK hg38 resource bundle (https://console.cloud.google.com/storage/browser/genomics-public-data/resources/broad/hg38/v0/), BWA–MEM GRCh38 reference genome (run ‘bwa.kit/run-gen-ref hs38DH’ from https://github.com/lh3/bwa/tree/master/bwakit), 1000 Genomes phase 3 (ftp://ftp-trace.ncbi.nih.gov/1000genomes/ftp/release/20110521), Ensembl v.105 (https://ftp.ensembl.org/pub/release-105/gtf/homo_sapiens/Homo_sapiens.GRCh38.105.gtf.gz), gnomAD v.2.1.1 (non-neuro) (https://gnomad.broadinstitute.org/variant/6-146865220-C-G?dataset=gnomad_r2_1_non_neuro), and Accelerating Medicines Partnership (AMP) program for PD (https://amp-pd.org/). Source data are provided with this paper.

## Code availability

All raw sequencing data were aligned to the GRCh38 reference genome using BWA–MEM (v.2.2.1) according to the pipeline described by Regier et al. (implementation can be found via GitHub at https://github.com/maarten-k/realignment (ref. ^42^) and via Zenodo at 10.5281/zenodo.10963076 (ref. ^43^). Joint genotyping was performed using a uniform pipeline according to the GATK best practices (v. 4.2.6.1). Handling and filtering of VCF files was performed using VCFtools (v. 0.1.16), BCFtools (v. 1.9) and PLINK (v. 1.90b6.21). Ancestry was estimated using LASER (v.2.04). Variants were annotated using Ensembl (GRCh38.105), snpEff (v.5.1d) and dbNSFP (v. 4.3a). Sample and variant quality control was performed using PLINK (v. 1.90b6.21) and RVAT (v. 0.2.0), while sample relatedness was inferred using KING (v. 2.2.7). All downstream analyses were performed using custom R code (performed in R 3.6.3) that we made available in the RVAT R package (v. 0.2.0) available via GitHub at https://github.com/kennalab/rvat (ref. ^53^) and via Zenodo at 10.5281/zenodo.10973472 (ref. ^57^). Other R packages used either as dependencies of RVAT or in other analyses and visualizations are ggplot2 (v. 3.4.2), ggrepel (v. 0.9.1), dplyr (v. 1.0.7), readr (v. 2.1.1), stringr (v. 1.4.0), tidyr (v. 1.1.4), magrittr (v. 2.0.1), kinship2 (v. 1.9.6), logistf (v. 1.25.0), SKAT (v. 2.2.5), SummarizedExperiment (v. 1.16.1), S4Vectors (v. 0.24.4), GenomicRanges (v. 1.38.0), IRanges (v. 2.20.2), libraDBI (v. 1.1.3) and RSQLite (v. 2.3.1). Sequence analysis for Sanger sequencing was performed using the v.29.0 PHRED/PHRAP/Consed software suite (http://www.phrap.org/) and variations in the sequences were identified via the Polyphred v6.15 software at http://droog.gs.washington.edu/polyphred/) (ref. ^54^).
